# Alcohol and Cannabis Use Trajectories and Outcomes in a Sample of Hispanic, White, and Asian Sexual and Gender Minority Emerging Adults

**DOI:** 10.3390/ijerph19042059

**Published:** 2022-02-12

**Authors:** Michael S. Dunbar, Daniel Siconolfi, Anthony Rodriguez, Rachana Seelam, Jordan P. Davis, Joan S. Tucker, Elizabeth J. D’Amico

**Affiliations:** 1 RAND Corporation, 4750 Fifth Avenue, Suite 600, Pittsburgh, PA 15213, USA; dsiconol@rand.org; 2 RAND Corporation, 20 Park Plaza, Suite 920, Boston, MA 02116, USA; anthonyr@rand.org; 3 RAND Corporation, 1776 Main Street, Santa Monica, CA 90401, USA; rseelam@rand.org (R.S.); jtucker@rand.org (J.S.T.); damico@rand.org (E.J.D.); 4 Suzanne Dworak-Peck School of Social Work, University of Southern California, 669 W 34th Street, Los Angeles, CA 90089, USA; jordanpd@usc.edu

**Keywords:** sexual minority, gender minority, race/ethnicity, substance use, trajectory analysis, emerging adulthood

## Abstract

Substance use disproportionately affects health and psychosocial outcomes for some racial/ethnic groups, but few longitudinal studies examine the extent to which sexual and gender minority (SGM) emerging adults of different racial/ethnic groups may experience disparities in outcomes at similar levels of alcohol or cannabis use. This study used five waves of annual survey data (spanning 2015 (average age 18) to 2020 (average age 23)) from an ongoing longitudinal cohort study of emerging adults. In the subset of 359 SGM emerging adults, separate sequelae of change models assessed differences in trajectories of alcohol or cannabis use (past 30-day frequency) and multiple health and psychosocial outcomes across Hispanic, Asian, and White individuals. White SGM emerging adults showed higher baseline levels of alcohol and cannabis frequency compared to Hispanic and Asian peers, but all groups showed similar rates of change (slope) over time. We observed few racial/ethnic differences in SGM emerging adult outcomes at the same levels of alcohol or cannabis use; that is, racial/ethnic groups showed similar patterns on most health and psychosocial outcomes; however, some differences emerged. For example, Asian respondents reported less engagement in sex with casual partners after using alcohol, marijuana, or other drugs compared to their White peers, at the same levels of alcohol use (β = −0.579, *p* = 0.03) or cannabis use (β = −0.737, *p* = 0.007). Findings underscore a need to consider multiple outcome domains and factors beyond additive stress in examining the effects of substance use across different groups of SGM individuals. More longitudinal studies with large, contemporary, and diverse samples of SGM emerging adults are needed to better characterize similarities and differences in patterns of substance use and use-related consequences in relation to intersecting SGM, racial/ethnic, and other identities.

## 1. Introduction

Research over the past several decades has identified mental health and substance use disparities among sexual and gender minority (SGM; e.g., gay, lesbian, bisexual, transgender) youth, adolescents, and emerging adults. These disparities are attributed to minority stressors experienced by SGM populations, including marginalization, discrimination, and victimization [1,2,3,4,5]. Much of the disparities-focused epidemiologic and theoretical research on SGM substance use has drawn from limited, specific subpopulations under the SGM umbrella (e.g., cisgender men who have sex with men) or has aggregated all SGM persons into a single group for comparisons with non-SGM peers. Recent years have highlighted the need to disaggregate the SGM pseudo-monolith [3,4,6,7]. For example, Minority Stress Theory [3,4,6] and the concept of intersectionality [1,8] may support an additive stress hypothesis, such that SGM persons of racial/ethnic minority status experience multiple layers of discrimination related to their sexual orientation, gender identity or expression, and/or their race and ethnicity. These additive stressors may, in turn, contribute to higher levels of substance use and poorer health or psychosocial outcomes. At the same time, as Schuler and colleagues have noted [9], empirical studies do not consistently support an additive stress perspective, and in some cases, persons with multiple minority identities (e.g., racial/ethnic minority identity and SGM identity) have similar or more favorable profiles of substance use or related outcomes than peers with fewer minority identities (e.g., non-Hispanic White SGM peers) [9]. Regardless of the direction of differences found in empirical studies, the social contexts at the intersections of sexual orientation, gender identity, race, and ethnicity contribute to unique lived experiences [1,10,11] that may be obscured when all SGM persons are grouped together and compared in aggregate to non-SGM peers. Thus, research needs to address these intersections to better understand whether and for whom substance use and related problems may be more severe among SGM individuals with diverse racial/ethnic identities.

To date, most research examining the additive stress hypothesis for racial/ethnic and SGM identities has used cross-sectional data and shown some within-group differences in outcomes. For example, in a study among emerging adult sexual minority women, Balsam et al. [12] found no racial/ethnic group differences for binge drinking and alcohol consequences, but did find that African American sexual minority young women reported lower peak drinking than White peers [12]. One longitudinal study [13] focused on sexual minority men found that White sexual minority men reported the highest initial levels of alcohol use compared to men with both racial/ethnic and sexual minority identities, but the patterns of change over time did not differ across racial/ethnic groups [13]. For cannabis use, Hispanic men reported higher initial levels and a greater increase than non-Hispanic White sexual minority men [13]. Finally, Schuler et al. [9] found that when comparing within-group racial/ethnic differences, Black and Hispanic sexual minority women had a greater magnitude of disparities than non-Hispanic White sexual minority women for cigarette smoking, heavy episodic drinking, and cannabis use [9]. Women of other racial identities or multiple racial identities also had greater disparities than non-Hispanic White women for cigarette smoking and cannabis use [9]. Thus, some data point to differences in patterns of substance use across racial/ethnic subgroups of SGM emerging adults, which may, in turn, increase the risk of negative outcomes for some individuals as they enter adulthood.

There are a number of gaps in the relatively limited body of research that has disaggregated SGM samples to explore differences across racial/ethnic groups. First, few studies use longitudinal data (e.g., tracking substance use from adolescence into emerging adulthood, when substance use typically peaks [14,15,16]). Second, few studies with disaggregated SGM samples have included Asian American and Pacific Islander SGM populations, who tend to be underrepresented in SGM research more generally [17,18]. Another gap in the extant literature relates to limited understanding of how substance use may differentially affect multiple domains of outcomes across SGM individuals with different racial/ethnic identities. For adolescents and emerging adults, substance use is associated with poorer outcomes across a range of domains relevant to holistic wellbeing, including educational attainment, employment, financial stability, justice system involvement, physical health, and behavioral health [19,20,21,22,23,24]. Similarly, substance use during the critical developmental period of emerging adulthood may hinder successful transitions to adult roles [22], which may set the stage for poorer outcomes across the lifespan. This underscores the importance of examining multiple outcome domains when characterizing potential differences for the effects of substance use across SGM emerging adults with differing racial/ethnic identities; however, most research has focused on substance use as the primary outcome, without consideration of other domains. Further, in studies using general population or community samples not restricted to SGM individuals, evidence suggests that racial and ethnic minority groups may experience poorer outcomes than non-Hispanic White peers at similar levels of substance use [25,26,27,28]. Thus, it is important to understand whether SGM emerging adults of different racial/ethnic groups may experience disparate outcomes that are not explained by any underlying differences in substance use. Additional studies with longitudinal samples of SGM individuals can help to shed light on the potential areas in which some SGM young people may or may not be differentially affected by substance use during the critical developmental period of emerging adulthood.

Recent research has shown that SGM emerging adults, compared as a whole to their non-SGM peers, experience disparate outcomes across a range of domains, even after adjusting for long-term alcohol and cannabis use trajectories [29]. Similarly, research has found that SGM youth experience worse school and juvenile justice sanctioning relative to their non-SGM peers, and these differences are not attributable to differences in delinquent behavior [30,31]. Findings emphasize the importance of addressing minority stressors and social and structural inequalities, which may be critical intervention targets to mitigate unmet behavioral health needs [29]. To our knowledge, there are virtually no longitudinal studies of contemporary SGM emerging adults that have simultaneously tested for racial and ethnic differences in outcomes after controlling for underlying substance use trajectories that may otherwise explain differential outcomes.

The current study aims to address several gaps in the existing literature on racial/ethnic differences in longitudinal patterns of alcohol and cannabis use during the period of youth to emerging adulthood and health and psychosocial outcomes among SGM individuals. Using longitudinal data from a contemporary cohort of SGM emerging adults, we examined trajectories of alcohol and cannabis use spanning the period of youth to emerging adulthood, stratified by race/ethnicity. After modeling these substance use trajectories, we tested whether there were differences across racial/ethnic groups in key outcomes spanning multiple domains (social relationships, education, economic wellbeing, physical health and mental health, and health care access). Significant differences would be indicative of racial/ethnic inequities in outcomes, above and beyond trajectories of substance use, which may be consistent with an additive stress hypothesis.

## 2. Materials and Methods

The study involved secondary analysis of survey data on a subset of SGM emerging adults from a longitudinal cohort study of emerging adults based in California [25,32]. Participants were originally recruited in 2008 (average age 11.5) from 16 middle schools in Southern California as part of a substance use prevention program (CHOICE) [32]. Inclusion criteria for the original CHOICE study included current attendance at one of the participating middle schools; youth were not excluded on the basis of substance use, future use risk, or other factors. Individuals completed survey waves 1 through 5 during middle school classes. After transitioning to high school after wave 5, participants were re-contacted and re-consented to complete annual web-based surveys and 61% of the wave 5 sample was retained at wave 6. Year-to-year retention rates for the web-based survey have ranged from 80% to 92%. At wave 12, fielded from June 2019 to July 2020, 2534 participants completed the survey. The current study uses data on a subset of 359 SGM emerging adults from waves 8 through 12 (corresponding to the end of high school to emerging adulthood). Study procedures were approved by the RAND Human Subjects Protection Committee.

### 2.1. Measures

#### 2.1.1. Alcohol and Cannabis Use Trajectories

Alcohol use and cannabis use at waves 8–12 were assessed using items from Monitoring the Future [33]: “During the past month, how many days did you (drink alcohol; use marijuana)?” Responses ranged from 0 to 20–30 days (note: beginning in Wave 11, response options changed to continuous 0 to 30 days).

#### 2.1.2. Sexual Orientation and/or Gender Minority Status

This study focused on the subset of individuals who met study criteria for SGM status at wave 12. Individuals were categorized as SGM if they met any of the following criteria, which were not mutually exclusive: self-reported sexual orientation of gay, lesbian, bisexual, or questioning; same-sex behavior; transgender identity; assigned sex at birth as intersex/other; reported a gender identity other than male or female; or if the respondent’s current gender identity was different from their sex assigned at birth (see [29]). Briefly, sexual orientation was assessed as: “Which of these best describes your sexual orientation?” (response options: Straight/heterosexual; Gay; Lesbian; Bisexual; Questioning; Asexual). Sexual behavior was assessed as “With whom of the following have you had vaginal or anal sex?” (response options: Only with females; Only with males; With both females and males). Assigned sex at birth was assessed by: “What was your sex assigned at birth?” (Male; Female; Intersex/Other). Current gender identity was assessed using the item “Do you currently identify as:” (Man, Woman, Gender Neutral [nonbinary], Other) and transgender identity was assessed with the item “Are you currently transgender?” (1 = yes; 0 = no).

#### 2.1.3. Racial/Ethnic Identity Groups

Individuals reported their race and ethnicity in response to “Which race/group best describes you? Mark all that apply” (response options: American Indian or Alaska Native, Native Hawaiian or Pacific Islander, Asian or Asian American, Black or African American, White or Caucasian, Other (another race or ethnicity)). Individuals are asked if they are Hispanic or Latino/Latina, and if so, what group best describes them (Central American, Cuban, Mexican, etc.; if not Hispanic or Latino/a, they would select “does not apply”). Race/ethnicity is determined as follows: those who endorse any of the Hispanic or Latino/a groups are categorized as being Hispanic or Latino/a (hereafter referred to as Hispanic); then, those who endorse being Asian are categorized as Asian if they do not also endorse any of the Hispanic groups; the White and Black groups comprise those respondents who only select White and Black, respectively, as the identity/group that best describes them. Those who are not already categorized as Hispanic or Asian and who select multiple groups or “Other” (another race or ethnicity) are categorized as multi-racial/other. 

To ensure adequate cell sizes to allow comparisons across groups, we focused on three racial/ethnic groups: White respondents who are not Hispanic (reference group; hereafter referred to as White; *n* = 107), Asian respondents who are not Hispanic (hereafter referred to as Asian; *n* = 64), and Hispanic respondents (*n* = 188). SGM respondents who identified as Black (*n* = 9), American Indian or Native Hawaiian (*n* = 2) and multi-race/another race (*n* = 22) were excluded from this analysis due to small cell sizes.

#### 2.1.4. Covariates

Model covariates included age, assigned sex at birth, and mother’s education (a proxy for socioeconomic status). We also included an indicator variable for CHOICE intervention status at wave 1 (note: the CHOICE intervention occurred in 2008–2009 and the intervention was not significantly associated with substance use or other outcomes after wave 2).

#### 2.1.5. Outcomes at Wave 12

*Education*. Post-high school education was assessed as a binary measure (0 = high school diploma or less; 1 = more than a high school diploma).

*Employment and economic stability*. Unemployment was assessed as a binary measure (0 = employed part-time or fulltime; 1 = unemployed). Additionally, participants reported whether they had been fired from a job in the past year (“In the past year, how often have you… been fired from a job?” (1 = Not at all to 6 = 20 or more times)). Response values were re-coded to represent the number of times the event occurred in the past year, with the mid-point taken of any response ranges (e.g., 6–9 times re-coded to 7.5); the range of the re-coded variable was 0–20. Respondents also reported on experiences of homelessness in the past year using the following items: “In the past 12 months, have you slept at a friend’s and/or family member’s home because you had nowhere else to stay (i.e., you did not have a fixed, stable nighttime residence at the time)?” (1 = yes, 0 = no) and “In the past 12 months, did you spend the night in any of these places because you had nowhere else to stay?” (e.g., A youth or adult shelter; In a public place, such as a train or bus station, a restaurant, or an office building; In an abandoned building; etc.). Items were summed and dichotomized to generate a single binary indicator of experiencing homelessness vs. not in the past 12 months. Food insecurity is assessed beginning in wave 12 as “how often in the past 12 months individuals experienced concerns with paying for food” (e.g., “I worried whether my food would run out before I got money to buy more”; “The food that I bought just didn’t last, and I didn’t have money to get more”) [34]. Response options were 1 = always true to 4 = never true. We created a dichotomous indicator such that those who endorsed any response other than “never true” on either item were coded as experiencing food insecurity.

*Transition to adult roles*. The IDEA short form [35] was used as a measure of perceived transition to independence/adulthood. Eight items (e.g., “This period of your life is a time of deciding on your own beliefs and values”) are measured on a 4-point Likert scale (response options: 1 = strongly disagree, 2 = somewhat disagree, 3 = somewhat agree, 4 = strongly agree). Total scores were calculated as the mean of all items with higher scores reflecting engagement in a greater number of emerging adult roles and a stronger perception of transitioning to adulthood.

*Criminal justice involvement*. Criminal justice involvement was assessed using a single item: “In the past year, how often have you… Gotten into trouble with the police because of something you did?” (1 = Not at all, 6 = 20 or more times, re-coded such that final variable has range 0–20).

*Social functioning*. Respondents completed the PROMIS Peer Relationships Short Form (e.g., “I was able to count on my friends”; response options: 0 = never to 4 = always) [36]. Items were summed and transformed to a t-score, with higher values indicating better social functioning. Loneliness was assessed by the 3-item UCLA Loneliness Scale [37]. Scores were summed, with higher values indicating greater loneliness.

*Physical health*. Self-reported physical health was assessed using a summary score based on the following items: perceived overall health (0 = excellent to 4 = poor), ability to physically engage in activities that one enjoys (1 = with no trouble to 5 = not able to do), and ability to participate in sports/activities similar to their peers (0 = with no trouble to 4 = not able to do) [38]. Items were reverse scored and summed, and higher scores indicated better physical health.

*Behavioral health*. Symptoms of depression were assessed with the 8-item Patient Health Questionnaire (PHQ-8) [39], symptoms of anxiety were assessed using the Generalized Anxiety Disorder (GAD-7) screen [40], and symptoms of post-traumatic stress disorder were assessed using the PCL-5 [41]. Sleep quality was assessed with a single item, “During the past month, how would you rate your overall sleep quality?” (response options: 1 = very good to 4 = very bad) [42].

*Sexual behavior*. Individuals reported the number of casual sexual partners (vaginal or anal sex) in the past three months. Participants also reported on risky sexual behavior using two separate items that assessed whether participants had sex with a casual partner after having used alcohol, marijuana, or other drugs in the past 3 months (yes/no) and whether they had sexual intercourse with a casual partner without using a condom (yes/no). Those who reported no casual sexual partners were coded as “no” for these two items.

*Unmet behavioral health treatment need*. Perceived unmet treatment need in the past year for alcohol and other drug use and mental health conditions were assessed using two items from the NSDUH [43]: “During the past 12 months, was there any time when you needed alcohol or any drug services or counseling for yourself but didn’t get it?” (1 = yes, 0 = no) and “During the past 12 months, was there any time when you needed mental health services or counseling for yourself but didn’t get it?” (1 = yes, 0 = no).

### 2.2. Analyses

Trajectories of alcohol and cannabis use were examined separately using latent growth modeling in a structural equation modeling framework with the weighted least squares with mean and variance adjusted estimator. We used a sequelae of change model [44], which allows the random effect of the rate of change of alcohol or cannabis use frequency to function as both an outcome and as a predictor of outcomes in emerging adulthood and allows multiple outcomes to be estimated simultaneously within the same model (i.e., vs. separate models for each dependent variable of interest), which yields better estimates of standard errors and provides a more accurate characterization of how predictors, such as alcohol or cannabis use and racial/ethnic group, affect various outcomes that may or may not be interrelated.

Model intercepts can be interpreted as the average frequency of alcohol or cannabis use at baseline (survey wave 8). Model slopes represent the change in frequency of alcohol or cannabis use from the end of high school to emerging adulthood (waves 8–12). All models were implemented in Mplus v8 [45].

To assess differences in trajectories across race/ethnicity groups, we first examined race/ethnicity (Hispanic, Asian, White (reference category)) as a predictor of the intercept and slope of alcohol or cannabis use frequency, controlling for age, sex assigned at birth, mother’s education, and intervention group. We then examined racial/ethnic differences in emerging adult outcomes (wave 12) by estimating models examining the direct effect from racial/ethnic group to each outcome, controlling for intercept and slope of alcohol or cannabis use predicting outcomes. This model can be interpreted as a test of differences in a given outcome (e.g., educational attainment) at wave 12 across racial/ethnic groups after accounting for trajectories of alcohol or cannabis use from waves 8–12 (i.e., at the same “level” of alcohol or cannabis use during emerging adulthood).

## 3. Results

### 3.1. Sample Characteristics

The final analytic sample included 359 SGM emerging adults. Sample descriptive characteristics are shown in Table 1. Briefly, participants in the analytic sample averaged approximately 18 years old at the first timepoint (wave 8) and 23 years old at wave 12, when health and psychosocial outcome data were reported. Approximately 64% identified as female (30% male; 6% gender neutral/nonbinary or another gender). The sample was approximately 30% White, 52% Hispanic, and 18% Asian.

### 3.2. Predictors of Intercept and Slope of Alcohol Use

**Figure 1:**
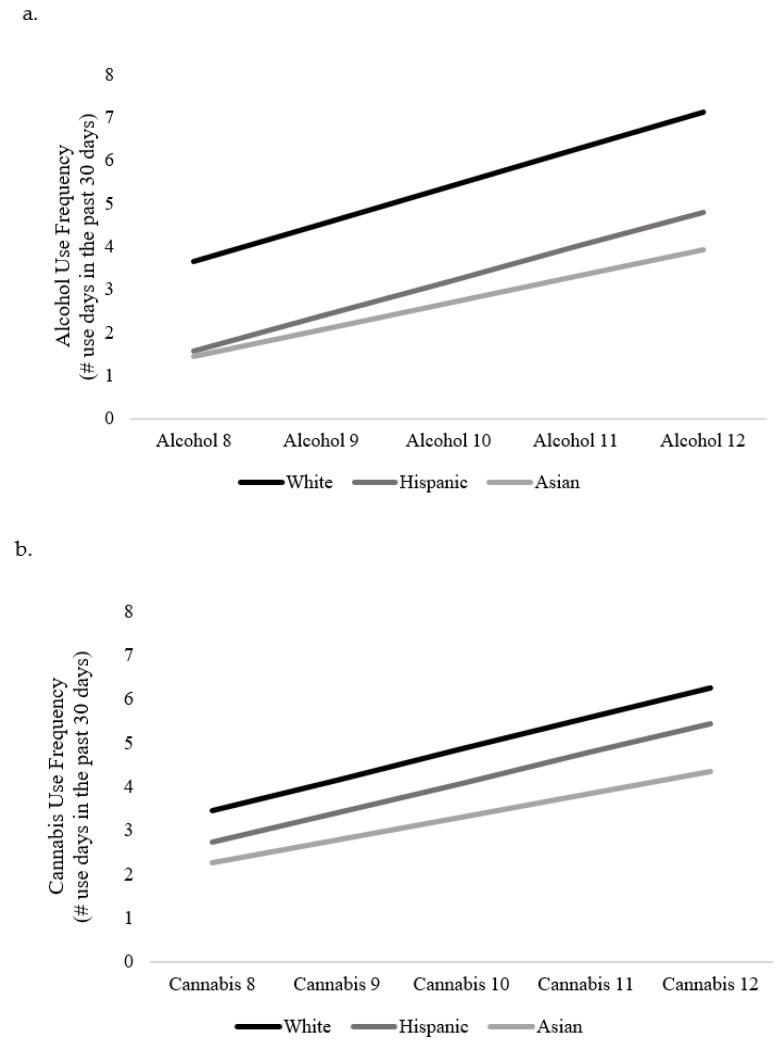
Trajectories of alcohol and cannabis use by racial/ethnic group. Panel (**a**) depicts the unconditional alcohol use frequency trajectories by racial-ethnic groups across waves 8 through 12. Panel (**b**) depicts the unconditional cannabis use frequency trajectories by racial-ethnic groups across waves 8 through 12.

The first model examined racial/ethnic differences in the intercept and slope for alcohol use frequency across waves 8–12, adjusting for age, sex, mother’s education, and intervention group. Overall model fit was acceptable: χ^2^ (28) = 81.801, RMSEA = 0.07, CFI = 0.90. Adjusting for covariates, there were significant differences by race/ethnicity on baseline frequency of alcohol use but not on the rate of change in alcohol use frequency over time. Specifically, emerging adults identifying as Hispanic (β = −0.510, *p* < 0.001) or Asian (β = −0.629, *p* < 0.001) reported a lower initial frequency of alcohol use compared to their non-Hispanic White peers. Trajectories by racial/ethnic group are presented in Figure 1a.

### 3.3. Racial/Ethnic Differences for Wave 12 Outcomes Controlling for Alcohol Use Trajectories

The next model examined direct effects from race/ethnicity to wave 12 outcomes, controlling for both the average (intercept) and rate of change (slope) of alcohol use frequency across waves 8 to 12, and can be interpreted as a test of the association between race/ethnicity and outcomes at wave 12 assuming both groups demonstrated the same trajectories (i.e., fixed intercept and slope) in the frequency of alcohol use over time (waves 8–12). The overall model fit was excellent (χ^2^ (110) = 164.073, RMSEA = 0.04, CFI = 0.97). Results for direct effects of race/ethnicity on outcomes, controlling for effects of alcohol use intercept and slope, are shown in Table 2. Full model results showing effects of use trajectory covariates (intercept; slope) on emerging adult outcomes are shown in supplemental Table S1. Briefly, greater average baseline frequency of alcohol use (intercept) was associated with higher social functioning scores (β = 0.165, *p* = 0.017) and greater likelihood of unmet treatment needs for alcohol or other drug use (β = 0.283, *p* < 0.001, Adjusted Odds Ratio (AOR) = 1.33), whereas rate of increase in drinking across waves (slope) was associated with greater frequency of sex with casual partners after using substances (β = 0.248, *p* = 0.04), higher IDEA scores (β = 0.224, *p* = 0.028), and lower likelihood of experiencing homelessness (β = −0.290, *p* = 0.038, AOR = 0.75).

At the same levels of use, emerging adults identifying as Hispanic reported a lower likelihood of post-high school educational attainment (β = −0.374, *p* = 0.017, AOR = 0.69) compared to White peers. Respondents identifying as Asian less frequently engaged in sex with casual partners after using alcohol, marijuana, or other drugs (β = −0.579, *p* = 0.03) compared to their White peers.

### 3.4. Predictors of Intercept and Slope of Cannabis Use

The first model examined racial/ethnic differences in the intercept and slope for cannabis use frequency across waves 8–12, adjusting for age, sex, mother’s education, and intervention condition. Model fit was good χ^2^ (28) = 50.16, RMSEA = 0.05, CFI = 0.97. With regard to initial cannabis use frequency (intercept), respondents identifying as Hispanic used cannabis less frequently (β = −0.303, *p* = 0.048) than respondents identifying as White. There were no racial/ethnic differences in the rate of change (slope) of cannabis use over time. Trajectories by racial/ethnic group are presented in Figure 1b.

### 3.5. Racial/Ethnic Differences for Wave 12 Outcomes Controlling for Cannabis Use Trajectories

We next examined direct effects from race/ethnicity to wave 12 outcomes, controlling for both the intercept and slope of cannabis use frequency across waves 8–12 along with age, sex at birth, mother’s education, and intervention condition. These models can be interpreted as a test of the association between race/ethnicity and outcomes at wave 12, assuming all groups demonstrated the same trajectories (i.e., fixed intercept and slope) of cannabis use frequency over time (waves 8–12). The overall model fit was excellent (χ^2^ (78) = 87.51, *p* = 0.22, RMSEA = 0.02, CFI = 0.99). Effects of cannabis use trajectory covariates (intercept; slope) on emerging adult outcomes are shown in Table S1. Briefly, similar to the alcohol models, higher baseline cannabis use frequency (intercept) was associated with greater perceived unmet treatment needs for alcohol or other drug use (β = 0.255, *p* = 0.047). With regard to slopes, increases in cannabis use frequency were associated with lower likelihood of post-high school educational attainment (β = −0.244, *p* = 0.006, AOR = 0.78) and lower likelihood of having perceived unmet treatment needs of AOD use (β = −0.262, *p* = 0.026, AOR = 0.77). 

Differences in outcomes by race/ethnicity at the same levels of cannabis use frequency (i.e., effects of race/ethnicity adjusting for cannabis trajectory intercept and slope) are shown in Table 3. Emerging adults identifying as Hispanic reported a lower likelihood of post-high school educational attainment (β = −0.405, *p* = 0.007, AOR = 0.67) and lower IDEA scores (β = −0.336, *p* = 0.006) than White peers. Respondents identifying as Asian reported less frequently engaging in sex with casual partners after alcohol, marijuana, or other drug use (β = −0.737, *p* = 0.007) and were less likely to have sex with a casual partner without using a condom (β = −0.589, *p* = 0.037, AOR = 0.55), compared to White peers.

## 4. Discussion

This study examined racial/ethnic group differences in alcohol/cannabis use trajectories over five years among SGM emerging adults across multiple health, social, and economic domains. This is one of the first papers to examine outcomes across a range of domains (social relationships, education, economic wellbeing, physical health and mental health, and health care access), especially after adjusting for longitudinal substance use as a risk factor for poorer outcomes. The study adds to the limited literature examining substance use and multiple domains of wellbeing among SGM samples disaggregated by racial/ethnic identities.

With respect to trajectories of alcohol and cannabis use, we observed racial/ethnic differences in baseline frequency of alcohol and cannabis use at the end of high school. Specifically, Hispanic- and Asian-identifying SGM emerging adults showed lower initial frequency of alcohol use compared to White individuals. Similarly, Hispanic individuals reported a lower initial frequency of past-month cannabis use compared to White SGM individuals; however, racial/ethnic groups were similar with respect to change in frequency of alcohol or cannabis use over time, which suggests that observed differences in baseline frequency of use may persist through emerging adulthood. This is consistent with previous research, which has shown that White emerging adult sexual minority men tend to report higher initial levels of use and continue to stay at higher levels over time [13]; however, in this same study, Halkitis and colleagues [13] reported differences in longitudinal patterns of cannabis use such that Hispanic emerging adult males had both higher initial levels of cannabis use and steeper increases over time than White men. Different patterns of findings across studies may be attributable to multiple factors, including differences in study samples, methods, and the time and place and policy environments in which data collection occurred. For example, Halkitis and colleagues used latent growth curve models to assess changes in the frequency of past-month substance use across 18 months among emerging adult sexual minority cisgender men, who were recruited in New York City between 2009 and 2011, when adult-use cannabis was not legal in that state. In contrast, the present study modeled change in substance use over a longer time span (approximately 5 years, between 2015 and 2020) in a more diverse cohort of SGM emerging adults based in California, where recreational cannabis became legal in 2018, and the sample was not limited to individuals identifying as cisgender men. It is possible that within SGM samples, racial/ethnic differences in longitudinal patterns of substance use may exist at the further intersections of sexual orientations (e.g., bisexual) and gender identities (e.g., nonbinary) that fall under the SGM umbrella [7,9,46].

At the same levels of alcohol or cannabis use (i.e., statistically adjusting for intercept and slope of alcohol or cannabis use), we observed few differences in emerging adult outcomes across racial/ethnic groups. One interpretation of this pattern of findings is that Hispanic, Asian, and White SGM individuals experience relatively similar outcomes across multiple domains at the same levels or alcohol or cannabis use. This could be viewed as a rejection of a strict, identity-based additive stress hypothesis, which would posit that stressors associated with intersecting identities (e.g., dual SGM identity and Hispanic or Asian identity) may contribute to poorer outcomes relative to White SGM peers, even at similar levels of substance use. Our results, save for a few exceptions, are consistent with previous studies that have shown relatively comparable outcomes among SGM subgroups with respect to some racial/ethnic identities. Although the evidence base is limited (particularly with respect to Asian SGM emerging adults), some previous studies have found few or no differences between racial/ethnic groups and others have shown that SGM persons of multiple minority identities fare better than peers with fewer minority identities (e.g., White SGM peers) [9]. This may be attributable to a range of factors. Firstly, racial/ethnic identity alone may provide limited insight into one’s lived experiences and frequency and severity of exposure to discrimination and other stressors; that is, racial/ethnic group identity is an imperfect proxy for individuals’ net exposure to stressors (note: our data did not include longitudinal information on stressors, such as experiences of racism). Similarly, one’s racial/ethnic group identity alone does not account for potential buffering or resiliency factors (e.g., peer, family, or community supports; coping strategies) that may protect against negative outcomes for some individuals. As such, a strict additive stress lens for interpreting racial/ethnic group differences (e.g., without consideration of other factors, including experienced stressors or resiliency) may not be appropriate for understanding within-SGM differences in substance use or related outcomes in relation to intersecting identities. In addition to measuring SGM identity, future survey research should also implement measures of discrimination and resiliencies.

It is important to note that, in comparison to non-SGM peers, SGM individuals experience greater discrimination, victimization, unmet needs, and evidence disparities in multiple domains [1,2,47,48]. For example, previous work by our group with this same cohort examined differences in outcomes between SGM and non-SGM individuals, finding consistent, significant disparities, such that SGM individuals showed poorer outcomes across nearly all domains [29]. In this context, the robust “main effect” of SGM status [49] may effectively mask potentially additive effects of racial/ethnic minority status on differences in outcomes within this subgroup.

We also note that, due to considerations of small sample size for some racial/ethnic subgroups, the present study focused on three racial/ethnic groups: White, Hispanic, and Asian. We could not, for example, assess potential differences for SGM individuals who identified as Black (*n* = 9 individuals in our sample) compared to other groups. Moreover, in addition to race/ethnicity, other potential intersectional characteristics for disaggregation include sexual orientation, gender identity, and socioeconomic status. Each of these are likely to shape the day-to-day lived experience of SGM populations and the stressors and supports encountered. Much of the existing SGM research has had relatively small sample sizes of SGM persons [2], which precludes further disaggregation. This was also the case in our analysis, and future studies with larger and more diverse samples will be critical for assessing whether and how findings from this study may or may not generalize to other racial/ethnic groups. More broadly, ongoing efforts to disaggregate the SGM umbrella are critical for providing a more nuanced characterization of the experiences and needs of diverse SGM individuals. Such information has important implications for informing culturally appropriate interventions and other efforts to improve equity and wellbeing for all SGM individuals.

We did observe some differences across racial/ethnic groups. Specifically, compared to White individuals at the same levels of alcohol or cannabis use, Hispanic-identifying SGM individuals reported a lower likelihood of post-high school education, and Asian individuals reported less frequent engagement in sex with casual partners after using drugs or alcohol. Additionally, at the same levels of cannabis use, Hispanic-identifying SGM emerging adults had significantly lower scores on an index of engagement in emerging adult roles compared to White SGM emerging adults. Asian-identifying SGM emerging adults were also significantly less likely than White SGM peers to endorse having sex with a casual partner without using a condom. This suggests that alcohol or cannabis use during the emerging adult years could disproportionately affect Hispanic identifying SGM emerging adults (versus White peers) with respect to educational attainment and the transition to adult roles. In contrast, Asian-identifying SGM emerging adults may experience better outcomes in some domains, even at similar levels of substance use, compared to White-identifying SGM peers. Findings underscore the importance of examining multiple outcome domains in efforts to characterize health and wellbeing, and potential within-group disparities, for SGM emerging adults.

As with all studies, findings must be considered in the context of limitations. First, due to very small cell sizes for some groups, we only examined differences across three racial/ethnic groups. As such, we cannot address potential differences between, for example, Black or multi-racial SGM emerging adults and White peers. This is an important limitation as past research has documented substance use disparities for Black and multiracial SGM adults [9]. Similarly, although a strength of the current study is its focus on Asian-identifying SGM emerging adults, our racial/ethnic groups are necessarily broad and do not account for important differences across subgroups. For example, Asian-identifying individuals represent a heterogeneous group, and past work has documented differences in risk factors for substance use across subgroups of Asian American adolescents [50]. Furthermore, we were unable to assess intersections between racial/ethnic identity and specific sexual orientations and/or gender identities (e.g., bisexual cisgender women, gay cisgender men, heterosexual transgender women, etc.). It is possible that, among SGM emerging adults, effects of racial/ethnic group identity on substance use and related outcomes may vary in relation to other important dimensions of SGM identity and lived experience (e.g., experienced stressors and buffers). Finally, the sample is predominantly from California, and therefore limited geographically. Future longitudinal studies with large and diverse samples from different geographic areas are needed to better understand the role of intersecting identities for SGM individuals on substance use trajectories and outcomes across the lifespan.

## 5. Conclusions

Overall, in this California-based sample, White SGM emerging adults showed higher levels of alcohol and cannabis frequency at the end of high school compared to Hispanic and Asian SGM peers, but all groups evidenced similar rates of change over time. We observed relatively few racial/ethnic differences in SGM emerging adult outcomes after adjusting for levels of alcohol or cannabis use over time. Given that other work has shown significant disparities for SGM emerging adults compared to their non-SGM peers at similar levels of substance use, and because some prior research has found disparate outcomes for minority groups even at similar levels of a risk factor (e.g., substance use), it was surprising to find few differences in outcomes in this sample. Findings add weight to the view that a simple “additive stress” orientation to intersectionality (based only on identity) is insufficient. Longitudinal work must continue to address potential differences in patterns of substance use and outcomes across multiple domains in relation to intersecting SGM, racial/ethnic, and other identities, but also lived experiences.

## Figures and Tables

**Table 1:** Sample characteristics.

	Full Analytic Sample (*n* = 359)	White (*n* = 107)	Hispanic (*n* = 188)	Asian (*n* = 64)	Group Difference
	% (n)/ Mean (SD)	% (n)/ Mean (SD)	% (n)/ Mean (SD)	% (n)/ Mean (SD)	*p*
Race/ethnicity					
White	29.8% (107)	--	--	--	--
Asian	17.8% (64)	--	--	--	
Hispanic	52.4% (188)	--	--	--	
Age (wave 8)	18.3 (0.8)	18.3 (0.7)	18.2 (0.8)	18.2 (0.8)	0.4717
Age (wave 12)	22.5 (0.8)	22.6 (0.7)	22.5 (0.8)	22.4 (0.7)	0.4222
Assigned Sex at Birth					
Male	32.0% (115)	29.9% (32)	33.0% (62)	32.8% (21)	0.8533
Female	68.0% (244)	70.1% (75)	67.0% (126)	67.2% (43)	
Intersex/other	0 (0)	0 (0)	0 (0)	0 (0)	
Gender Identity					
Man	29.8% (107)	27.1% (29)	31.9% (60)	28.1% (18)	0.3105
Woman	64.4% (231)	62.6% (67)	63.8% (120)	68.8% (44)	
Gender neutral	4.2% (15)	6.5% (7)	3.2% (6)	3.1% (2)	
Another identity	1.7% (6)	3.7% (4)	1.1% (2)	0 (0)	
Mother’s Education					
<High school	16.2% (58)	1.9% (2)	28.7% (54)	3.1% (2)	<0.0001
High school	15.6% (56)	12.2% (13)	18.1% (34)	14.1% (9)	
Some college	20.6% (74)	22.4% (24)	21.8% (41)	14.1% (9)	
Associate’s degree	7.0% (25)	7.5% (8)	6.9% (13)	6.3% (4)	
College degree or higher	37.1% (133)	52.3% (56)	20.7% (39)	59.4% (38)	
Don’t know	3.6% (13)	3.7% (4)	3.7% (7)	3.1% (2)	
SGM characteristics ^a^					
Sexual orientation					
Straight/heterosexual	8.4% (30)	6.5% (7)	7.5% (14)	14.1% (9)	0.2314
Gay	14.2% (51)	11.2% (12)	16.5% (31)	12.5% (8)	
Lesbian	9.8% (35)	14.0% (15)	8.5% (16)	6.3% (4)	
Bisexual	57.1% (205)	57.0% (61)	59.0% (111)	51.6% (33)	
Questioning	10.6% (38)	11.2% (12)	8.5% (16)	15.6% (10)	
Asexual	0 (0)	0 (0)	0 (0)	0 (0)	
Same-sex vaginal/anal sex	47.1% (168)	41.5% (44)	52.9% (99)	39.1% (25)	0.0623
Different gender identity vs. sex assigned at birth	8.4% (30)	15.9% (17)	4.3% (8)	7.8% (5)	0.0024
Gender neutral or other gender identity	5.9% (21)	10.3% (11)	4.3% (8)	3.1% (2)	0.1937
Transgender identity	2.8% (10)	6.5% (7)	0.5% (1)	3.1% (2)	0.0104
Intersex/other	0 (0)	0 (0)	0 (0)	0 (0)	--

Note: Group differences assessed by unadjusted bivariate *t*-test and omnibus chi-square tests. SGM = sexual/gender minority. ^a^ Not mutually exclusive.

**Table 2:** Parameter estimates of race/ethnicity predicting wave 12 outcomes, controlling for alcohol use trajectories.

		Hispanic (Reference Group Is White)	Asian (Reference Group Is White)
Domain	Outcome Variable	Standardized Beta (95% CI) *p*-Value	Standardized Beta (95% CI) *p*-Value
*Education*	Educational attainment post-high school (yes)	−0.374 (−0.682, −0.066) *p* = 0.017	0.070 (−0.359, 0.499) *p* = 0.749
*Employment and economic stability*	Unemployed full- or part-time (yes)	0.069 (−0.282, 0.421) *p* = 0.699	0.039 (−0.384, 0.461) *p* = 0.857
	Number of times fired from job in past year	0.141 (−0.305, 0.587) *p* = 0.535	0.009 (−0.560, 0.578) *p* = 0.975
	Experienced homelessness (yes)	−0.348 (−0.736, 0.041) *p* = 0.079	−0.332 (−0.875, 0.211) *p* = 0.231
	Experienced food insecurity (yes)	0.126 (−0.190, 0.443) *p* = 0.434	−0.133 (−0.546, 0.279) *p* = 0.526
*Transition to adult roles*	IDEA scale	−0.272 (−0.568, 0.024) *p* = 0.072	−0.040 (−0.405, 0.324) *p* = 0.828
*Criminal justice involvement*	Instances of being in trouble with police in past year	0.096 (−0.338, 0.530) *p* = 0.665	0.164 (−0.333, 0.662) *p* = 0.517
*Social functioning*	PROMIS Social Functioning score	0.094 (−0.161, 0.349) *p* = 0.472	0.147 (−0.181, 0.475) *p* = 0.379
	Loneliness score	−0.164 (−0.442, 0.114) *p* = 0.247	−0.122 (−0.457, 0.212) *p* = 0.474
*Physical health*	Physical health score	−0.172 (−0.436, 0.091) *p* = 0.200	−0.193 (−0.536, 0.150) *p* = 0.271
*Behavioral health*	Anxiety—GAD-7 score	0.043 (−0.231, 0.318 ) *p* = 0.758	−0.145 (−0.485, 0.196) *p* = 0.405
	Depression—PHQ 8 score	0.037 (−0.241, 0.315) *p* = 0.793	−0.076 (−0.430, 0.279) *p* = 0.675
	PTSD—PCL-5 score	0.025 (−0.355, 0.406) *p* = 0.897	0.137 (−0.433, 0.706) *p* = 0.638
	Sex with casual partner after using alcohol, marijuana, or other drugs (yes)	−0.067 (−0.423, 0.289) *p* = 0.712	−0.579 (−1.102, −0.056) *p* = 0.030
	Sex with casual partner without condom (yes)	0.087 (−0.302, 0.475) *p* = 0.662	−0.508 (−1.079, 0.064) *p* = 0.082
	Sleep quality	0.042 (−0.223, 0.306) *p* = 0.758	−0.105 (−0.457, 0.247) *p* = 0.560
	Number of casual sex partners	0.101 (−0.371, 0.572) *p* = 0.676	−0.012 (−0.516, 0.493) *p* = 0.963
*Unmet treatment need*	Unmet treatment need for alcohol or other drug use (yes)	−0.019 (−0.543, 0.505) *p* = 0.943	−0.195 (−0.950, 0.560) *p* = 0.613
	Unmet treatment need for mental health (yes)	−0.074 (−0.396, 0.249) *p* = 0.655	−0.130 (−0.554, 0.294) *p* = 0.549

Note: Values are standardized parameter estimates and 95% CI (lower, upper) and *p* values. Separate models assessed direct effects of race/ethnicity on each outcome, controlling for age, assigned sex at birth, mother’s education, and intervention group at wave 8.

**Table 3:** Parameter estimates of race/ethnicity predicting wave 12 outcomes, controlling for cannabis use trajectories.

		Hispanic (Reference Group Is White)	Asian (Reference Group Is White)
Domain	Outcome Variable	Standardized Beta (95% CI) *p*-Value	Standardized Beta (95% CI) *p*-Value
*Education*	Educational attainment post-high school (yes)	−0.405 (−0.699, −0.112) *p* = 0.007	−0.007 (−0.388, 0.374) *p* = 0.971
*Employment and economic stability*	Unemployed full- or part-time (yes)	0.039 (−0.298, 0.376) *p* = 0.820	−0.003 (−0.407, 0.401) *p* = 0.990
	Number of times fired from job in past year	0.169 (−0.080, 0.418) *p* = 0.183	0.043 (−0.267, 0.353) *p* = 0.788
	Experienced homelessness (yes)	−0.303 (−0.678, 0.072) *p* = 0.113	−0.261 (−0.755, 0.232) *p* = 0.299
	Experienced food insecurity (yes)	0.239 (−0.061, 0.539) *p* = 0.119	−0.001 (−0.400, 0.397) *p* = 0.994
*Transition to adult roles*	IDEA scale	−0.336 (−0.577, −0.095) *p* = 0.006	−0.132 (−0.436, 0.172) *p* = 0.395
*Criminal justice involvement*	Instances of being in trouble with police in past year	0.096 (−0.155, 0.346) *p* = 0.455	0.167 (−0.144, 0.477) *p* = 0.293
*Social functioning*	PROMIS Social Functioning score	−0.003 (−0.252, 0.247) *p* = 0.983	0.017 (−0.293, 0.328) *p* = 0.913
	Loneliness score	−0.139 (−0.390, 0.112) *p* = 0.277	−0.104 (−0.415, 0.208) *p* = 0.514
*Physical health*	Physical health score	−0.171 (−0.420, 0.079) *p* = 0.180	−0.190 (−0.500, 0.120) *p* = 0.230
*Behavioral health*	Anxiety—GAD-7 score	−0.009 (−0.260, 0.242) *p* = 0.945	−0.217 (−0.528, 0.094) *p* = 0.171
	Depression—PHQ 8 score	0.017 (−0.233, 0.268) *p* = 0.893	−0.107 (−0.418, 0.204) *p* = 0.502
	PTSD—PCL-5 score	−0.181 (−0.511, 0.149) *p* = 0.282	0.038 (−0.445, 0.520) *p* = 0.878
	Sex with casual partner after using alcohol, marijuana, or other drugs (yes)	−0.190 (−0.538, 0.158) *p* = 0.285	−0.737 (−1.268, −0.206) *p* = 0.007
	Sex with casual partner without condom (yes)	0.047 (−0.332, 0.427) *p* = 0.806	−0.589 (−1.141, −0.037) *p* = 0.037
	Sleep quality	0.035 (−0.213, 0.284) *p* = 0.780	−0.110 (−0.419, 0.199) *p* = 0.486
	Number of casual sex partners	0.118 (−0.135, 0.372) *p* = 0.359	−0.003 (−0.317, 0.310) *p* = 0.983
*Unmet treatment need*	Unmet treatment need for alcohol or other drug use (yes)	−0.104 (−0.620, 0.413) *p* = 0.694	−0.371 (−1.101, 0.360) *p* = 0.320
	Unmet treatment need for mental health (yes)	−0.199 (−0.507, 0.108) *p* = 0.203	−0.293 (−0.701, 0.116) *p* = 0.160

Note: Values are standardized parameter estimates and 95% CI (lower, upper) and *p* values. Separate models assessed direct effects of race/ethnicity on each outcome, controlling for age, assigned sex at birth, mother’s education, and intervention group at wave 8.

## Data Availability

Data are not available as the parent study is still in progress.

## Supplementary Materials

The following supporting information can be downloaded at: https://www.mdpi.com/article/10.3390/ijerph19042059/s1, Table S1: Parameter estimates of alcohol and cannabis use predicting wave 12 outcomes.
