# Supplementary material for: Alcohol and Cannabis Use Trajectories and Outcomes in a Sample of Hispanic, White, and Asian Sexual and Gender Minority Emerging Adults
Source: Int J Environ Res Public Health. 2022 Feb 12;19(4):2059. doi: 10.3390/ijerph19042059 (PMC8871829; doi:10.3390/ijerph19042059)
Supplement: Supplementary file 1 [file ijerph-19-02059-s001.zip › ijerph-1568805-supplementary.pdf]

Supplemental Table S1. *Parameter estimates of alcohol and cannabis use trajectories predicting wave 12 outcomes*

	**Effect of alcohol use trajectory (waves 8-12)**		**Effect of cannabis use trajectory (waves 8-12)**	
**Outcome variable**	*Intercept* β (95% CI [LL, UL])	*Slope* β (95% CI [LL, UL])	*Intercept* β (95% CI [LL, UL])	*Slope* β (95% CI [LL, UL])
Educational attainment post-high school (yes)	0.083 (-0.073, 0.238) p=.297	-0.090 (-0.305, 0.125) p=.414	0.041 (-0.137, 0.219) p=.651	**-0.244 (-0.416, -0.072) p=.006**
Currently unemployed (yes)	0.043 (-0.140, 0.225) p=.647	-0.072 (-0.311, 0.166) p=.554	-0.044 (-0.228, 0.141) p=.641	-0.095 (-0.296, 0.106) p=.352
Number of times fired from job in past year	0.032 (-0.066, 0.130) p=.518	-0.112 (-0.312, 0.089) p=.275	0.076 (-0.066, 0.219) p=.294	-0.024 (-0.169, 0.121) p=.750
Experienced homelessness in past year (yes)	0.010 (-0.172, 0.193) p=.913	**-0.290 (-0.564, -0.016) p=.038**	0.019 (-0.231, 0.269) p=.881	-0.019 (-0.235, 0.196) p=.862
Experienced food insecurity in past year (yes)	-0.081 (-0.261, 0.098) p=.374	-0.134 (-0.370, 0.101) p=.264	0.151 (-0.014, 0.315) p=.073	0.004 (-0.184, 0.192) p=.966
IDEA scale	0.063 (-0.046, 0.172) p=.254	**0.224 (0.025, 0.423) p=.028**	0.026 (-0.111, 0.163) p=.714	0.118 (-0.018, 0.253) p=.088
Instances of being in trouble with police in past year	.088 (-0.013, 0.190) p=.088	-0.188 (-0.399, 0.023) p=.080	0.056 (-0.087, 0.199) p=.445	-0.059 (-0.204, 0.085) p=.422
PROMIS Social Functioning score	**0.165 (0.030, 0.300) p=.017**	0.061 (-0.121, 0.244) p=.510	0.032 (-0.104, 0.168) p=.648	-0.015 (-0.151, 0.121) p=.826
Loneliness score	-0.027 (-0.164, 0.109) p=.694	0.015 (-0.174, 0.204) p=.877	-0.012 (-0.148, 0.125) p=.867	-0.092 (-0.227, 0.044) p=.185
Physical health score	-0.025 (-0.145, 0.095) p=.687	0.001 (-0.177, 0.179) p=.991	-0.037 (-0.175, 0.101) p=.601	-0.055 (-0.192, 0.083) p=.436
Anxiety - GAD-7 score	0.059 (-0.082, 0.200) p=.414	0.093 (-0.101, 0.287) p=.346	-0.009 (-0.148, 0.129) p=.895	-0.025 (-0.162, 0.112) p=.720
Depression - PHQ 8 score	-0.023 (-0.150, 0.105) p=.726	0.128 (-0.050, 0.305) p=.159	-0.045 (-0.183, 0.092) p=.519	0.002 (-0.135, 0.139) p=.977

PTSD - PCL-5 score	0.034 (-0.146, 0.213) p=.713	0.202 (-0.043, 0.447) p=.105	-0.063 (-0.243, 0.116) p=.490	0.147 (-0.028, 0.322) p=.100
Sex with casual partner after using alcohol, marijuana, or other drugs (yes)	0.171 (-0.008, 0.349) p=.061	**0.248 (0.011, 0.485) p=.040**	0.041 (-0.142, 0.223) p=.663	0.135 (-0.045, 0.315) p=.141
Sex with casual partner without condom (yes)	0.067 (-0.130, 0.265) p=.504	0.082 (-0.192, 0.357) p=.556	0.062 (-0.123, 0.247) p=.511	-0.129 (-0.288, 0.030) p=.111
# casual sexual partners	-0.045 (-0.255, 0.166) p=.678	0.074 (-0.206, 0.353) p=.605	-0.019 (-0.154, 0.115) p=.777	0.019 (-0.119, 0.157) p=.787
Sleep problems	-0.046 (-0.180, 0.089) p=.507	0.022 (-0.161, 0.204) p=.817	-0.106 (-0.242, 0.031) p=.128	-0.082 (-0.218, 0.054) p=.237
Unmet treatment need for alcohol or other drug use (yes)	**0.283 (0.125, 0.441) p<.001**	-0.124 (-0.396, 0.149) p=.374	**0.255 (0.004, 0.505) p=.047**	**-0.262 (-0.492, -0.031) p=.026**
Unmet treatment need for mental health (yes)	0.127 (-0.051, 0.306) p=.162	0.085 (-0.157, 0.328) p=.491	-0.117 (-0.311, 0.078) p=.239	-0.091 (-0.261, 0.080) p=.298

Note. Values are standardized estimates of the direct effect of intercept and slope of past-month alcohol use or cannabis use trajectories from waves 8 to 12 from separate sequelae of change models. Models controlled for age, assigned sex at birth, race/ethnicity, mother's education, and intervention group at wave 1. β = standardized model effect estimate. CI = confidence interval; LL = lower limit; UL = upper limit. PTSD = Post-traumatic stress disorder.
